# A Review of Flood-Related Storage and Remobilization of Heavy Metal Pollutants in River Systems

**DOI:** 10.1007/s11270-016-2934-8

**Published:** 2016-06-22

**Authors:** Dariusz Ciszewski, Tomáš Matys Grygar

**Affiliations:** 1 AGH University of Sciences and Technology, Krakow, Poland; 2 Institute of Inorganic Chemistry, AS CR, v.v.i., Řež, Czech Republic; 3 Faculty of Science, J.E. Purkyně University in Ústí nad Labem, Ústí nad Labem, Czech Republic

**Keywords:** River, Sediment, Heavy metals, Mobilization, Pollution, Flood

## Abstract

Recently observed rapid climate changes have focused the attention of researchers and river managers on the possible effects of increased flooding frequency on the mobilization and redistribution of historical pollutants within some river systems. This text summarizes regularities in the flood-related transport, channel-to-floodplain transfer, and storage and remobilization of heavy metals, which are the most persistent environmental pollutants in river systems. Metal-dispersal processes are essentially much more variable in alluvia than in soils of non-inundated areas due to the effects of flood-sediment sorting and the mixing of pollutants with grains of different origins in a catchment, resulting in changes of one to two orders of magnitude in metal content over distances of centimetres. Furthermore, metal remobilization can be more intensive in alluvia than in soils as a result of bank erosion, prolonged floodplain inundation associated with reducing conditions alternating with oxygen-driven processes of dry periods and frequent water-table fluctuations, which affect the distribution of metals at low-lying strata. Moreover, metal storage and remobilization are controlled by river channelization, but their influence depends on the period and extent of the engineering works. Generally, artificial structures such as groynes, dams or cut-off channels performed before pollution periods favour the entrapment of polluted sediments, whereas the floodplains of lined river channels that adjust to new, post-channelization hydraulic conditions become a permanent sink for fine polluted sediments, which accumulate solely during overbank flows. Metal mobilization in such floodplains takes place only by slow leaching, and their sediments, which accrete at a moderate rate, are the best archives of the catchment pollution with heavy metals.

## Background

Economic development, which has rapidly grown since the Industrial Revolution, has been accompanied by an increasing demand for heavy metals and substances containing metal compounds. Heavy metals escape during ore extraction and processing and are also widespread in industrial and municipal sewages; their sources have been extensively reviewed in earlier works (e.g. Förstner and Wittmann 1981; Salomons and Förstner 1984). For almost 200 years of extensive metal utilization, the possible toxic effects of the intake of heavy metals were not recognized, and industrialization has resulted in their uncontrolled dispersal in hundreds of kilometres of river systems.

Heavy metals are discharged in both dissolved and solid phases in proportions that vary greatly depending on the element properties, pollution sources and chemistry of receiving river waters. In river water, metals tend to precipitate rapidly or to be adsorbed onto solid particles. These processes may be reversed with changes in the Eh and pH, which are master variables that control the partitioning of metals between sediments and the water column (Salomons and Förstner 1984). In river systems, concentrations of the same metal in fine-grained sediment can be one to several orders of magnitude higher than in the dissolved phase (Martin and Meybeck 1979; Horowitz 1991). Freshly deposited sediments may easily liberate metals during resuspension with an increase of flow velocity (Salomons et al. 1987). A fine fraction of sediment, comprising silt and clay, has been recognized as the crucial component of the pollution load stored within many river systems (Owens et al. 2005), whereas heavy metals associated with coarse-grained sediments constitute an important part of the pollution load over short reaches of some mine-affected rivers (Marron 1989; Ciszewski 1998). Conventionally, the part of metals, which passes through 0.45-μm filter, is named “dissolved”, although it does not represent truly dissolved metal ions. It is composed of free metals, complexions or metals bounds to ligands, which may aggregate into organic or inorganic entities of colloidal size (1.2 μm–1 kDa). Metals associated with colloids cannot settle themselves until aggregated into larger particles and can be conveyed with dissolved phase over long distances (Gueguen and Dominik 2003).

Sediment-associated heavy metals can be stored in fluvial systems for periods from days to millennia, depending on the river-flow dynamics. It has been estimated that river channels of mid-sized catchments, of the order of thousands square kilometres, are responsible for the storage of only a small percentage of the annual sediment-associated pollutant flux (Walling et al. 2003; Villarroel et al. 2006). The storage of fine-grained sediment in channelized urban rivers is of the order of days to months (Taylor and Owens 2009). In channels of less modified rivers, sediments of fine-grained sand and silt fractions can be stored for several to tens of years in shelters, at confluences or in wide channel sections as sediment “plugs” and sequences many decimetres thick (Skalak and Pizzuto 2010; Faměra et al. 2013). For most perennial river systems, overbank sedimentation is considered to represent longer-term storage for fine sediments with a much larger residence time of the order of 10^2^–10^3^ years (Matys Grygar et al. 2016a), representing a net loss to actual downstream sediment conveyance (Walling et al. 1999). The floodplain pollutant flux depends on sediment deposition, which is typically a few tens of percentage points of annual sediment load delivered to the main channel system. It depends on the actual sediment budget; e.g., in the mid-sized catchment of the River Aire, approximately one third of historical anthropogenic Pb has been stored in the floodplain (Walling et al. 2003).

Floods are known both for their devastating potential of human infrastructure and for maintaining valuable riparian communities. They also play a crucial role in creating and reshaping the dispersal pollutant patterns in a river system (Matys Grygar et al. 2014). During floods, the pollutants formerly temporarily stored in the channel are quickly entrained and transferred to the floodplain (Matys Grygar et al. 2014). In turn, the erosion and leaching of the polluted floodplain sediments was recognized as important factor influencing secondary river pollution proportional to the degree of historical pollution and dilution by the enhanced input of particulates from the watershed and bank erosion (Navrátil et al. 2008; Chen et al. 2014). Floods may also trigger primary pollution when precipitation extremes cause failures of settling ponds or washes from stockpiles. A special case of an artificially enhanced discharge-driven pattern is a “pollution pulse”—a sediment wave or sediment slug—the introduction of extra material, e.g. due to mining operations (Miller et al. 1998), which is then only slowly further transported downstream by the trunk river.

Given that previous reviews have considered impact of metal mining on the aquatic environment (Byrne et al. 2012; Wolkersdorfer 2004; Miller 1997) or essentially have summarized the effects of mining on the rate of natural fluvial processes and contamination along mine-affected rivers (Macklin 1996), this review focus on artificially modified rivers, which have been usually contaminated from numerous point sources. Such rivers are widespread in densely populated and industrialized areas of the world and characterized by fluvial processes altered due to engineering structures. Particular attention is given to floods and we stress that floods and high-water stages are the reasons, which speed up metal circulation in river valleys by orders of magnitude if compared to the non-inundated soils. Unlike previous reviews (Du Laing et al. 2009; Schultz-Zunkel and Krueger 2009), we do not deal with estuaries with salinity effects on metal mobility and give more attention to fluvial processes as they have principal effects on metal dispersal. We review the effects of floods and high-water stages on the dispersal of heavy metals in channels and floodplains in seven main sections. We start with the description of the hydraulic control of heavy-metal storage in a channel (Section 2), modes of channel-to-floodplain heavy-metal transfer (Section 3), the longitudinal and spatial patterns of floodplain storage (Section 4), the influence of channel engineering on heavy-metal storage (Section 5), the role of impoundments in the storage of heavy metals (Section 6), the influence of floods on metal remobilization (Section 7) and methods of pollution mapping (Section 8).

## Flood Control on Heavy Metals’ Storage in a Channel

Floods play the most important role in the transport of heavy-metal pollutants associated with particulate matter, particularly in severely polluted catchments. In such river systems, both concentrations of suspended particulate matter and pollutant contents increase with the growing discharge, particularly in the early stage of floods. Their values sometimes remain high even during the flood attenuation (Müller and Wessels 1995; Baborowski et al. 2004; Coynel et al. 2007a, b; Resongles et al. 2015), producing a counter-clockwise hysteresis loop of total pollutant loads versus discharge (Zonta et al. 2005; Coynel et al. 2007a, b). The loop documents the activation of pollutants and sediments as enhanced runoff and river discharge pass through their temporary sink. Higher discharges may also increase dissolved pollutant concentrations, e.g., in the case of As; by contrast, the dissolved concentrations of most other pollutants, i.e., those primarily transported in particulate forms, are decreased due to dilution by excess water (Baborowski et al. 2004; Resongles et al. 2015).

It was found that periods of low flows in dry seasons lead to higher concentrations of heavy metals in channel-bed sediments, whereas wet seasons are characterized by a lower metal content in the bed and a higher metal content in suspended sediments (Gaiero et al. 1997; He et al. 1997). Physical seasonal changes may be exacerbated by changing redox conditions at the river bottom, associated with varying organic matter and Fe- and Mn-oxide contents in sediments (Gaiero et al. 1997; He et al. 1997). Floods may also change the river-water chemistry. In acidic waters, such as those containing acid-mine drainage, the enhanced input of rainwater may dilute excess acidity and promote the hydrolysis of Fe^3+^ ions, followed by the precipitation of Fe oxides and sweeping a part of dissolved heavy metals to solid particles (Cánovas et al. 2012).

Floods shift the boundary between bedload and suspended load by re-suspending the fine sediment fractions from their temporary sinks in the channel. The storage of sediment-associated heavy metals in a channel is a complex interplay of erosion, accumulation and sediment reworking. In a given cross section, the intensity of these processes depends on the turbulence and velocity of current flow, which is a function of the channel shape and the bed morphology producing remarkable spatial heterogeneity. In a sand-bed alluvial, perennial river, the current flow intensity is reflected in metal content in a fraction <1 mm (Ciszewski 1998). Metal content in fine silt and clay, <0.063-mm fraction, reflects the metal concentration observed in a suspended load. The fine-grained fraction accumulates primarily in the shelters near channel banks, where the flow velocity enables the settling of small and low-density particles (Rhoads and Cahill 1999). A part of fine-grained sediments is also trapped from suspension by plants and plant roots producing homogeneous fine-grained sediments, best suitable for river monitoring (Ciszewski 1998; Nováková et al. 2013). The lowest heavy-metal concentrations usually occur in channel bars; however, on a gravel bed river, polluted fines can infiltrate in greater amounts into gravel substrate during low-water stages (Ladd et al. 1998). Furthermore, being the deepest places in the channel, pools can be a place to store fine sediments during low-water stages and for the precipitation of manganese and iron hydroxides, with some metals, on gravel surfaces (Evans and Davies 1994). During floods, sediment-associated heavy metals are scoured from pools (Ciszewski 1997). On the contrary, in channels of episodic rivers, the maximal concentrations occur in the zone of the most frequent flow, and much smaller concentrations are in the near-bank deposits (Graf et al. 1991). The complexity of the channel dynamics and the transient nature of channel sediments make their stratigraphy too complicated to reconstruct the pollution history (Nováková et al. 2013; Faměra et al. 2013).

In river reaches with permanently active pollution sources, the effect of flushing of polluted, fine-grained sediments by floods is limited, and metal concentrations can quickly return to the previous state. On the Biała Przemsza River, concentrations of zinc, lead and cadmium dropped approximately 3- to 4-fold following a 100-year flood over a 40-km reach downstream the discharge point of effluents from the lead–zinc ore mine in southern Poland (Ciszewski 2001). The drop was accompanied by the coarsening of near-bank channel deposits. In the reach adjacent to the pollution source, over the year following the flood, concentrations of metals increased, whereas downstream reaches exhibited further decreases in concentrations. On reaches at former mine sites, overbank sediments contribute to the pollution of the channel, delaying pollution decrease by natural dilution with extra clean material. In some cases, it is estimated that natural decay of metals within a river channel may last several hundred years (Moore and Langner 2012). On the Carson River in Nevada, pollution of the channel sediment was not markedly altered even by 100-year flood due to the mobilization of polluted overbank sediments (Miller et al. 1999). The long-term stability of channel-sediment pollution is also observed on small perennial streams despite numerous floods passing through a river system. Investigations of the Matylda stream sediments pollution by heavy metals indicated only a small decrease over 40 years after mining cessation (Ciszewski et al. 2012).

**Fig. 1:**
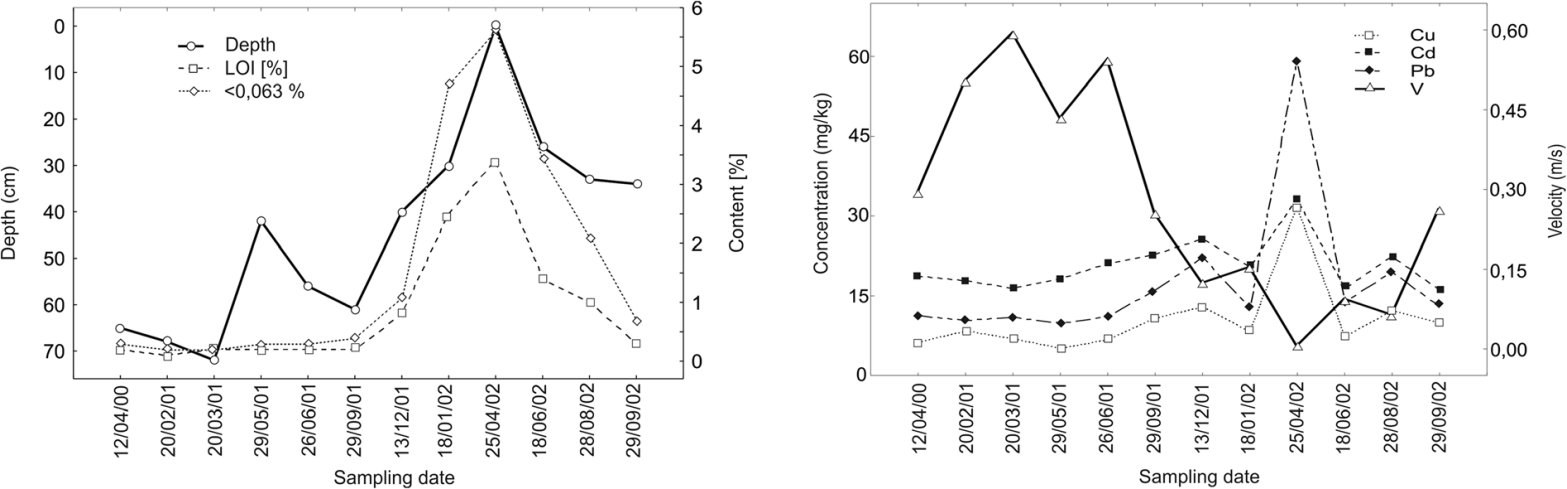
Changes of heavy metals concentrations, losses on ignition, content of fine fraction and water depth in the same place of the channel are related to channel bar migration during the 2 years period (from Ciszewski 2004, modified)

Nevertheless, the heavy-metal dispersal pattern in channel sediments can change in particular cross-sections after flood flows, and concentrations can drop, go up and stay the same in particular channel locations (Protasowicki et al. 1999; Moody et al. 2000). On the Mała Panew River, incised in sandy alluvia, pollutant (Ba and Zn) concentrations were observed for 2 years in the same channel locations (Ciszewski 2004). In these places, the migration of sandbars several metres long and several tens of decimetres high resulted from the passing of two floods of moderate magnitudes. Usually, a dead zone, characterized by flow velocities below <0.1 m/s, appeared in front of the bar at low discharges. It occurred for a few months until it was eroded or filled with the sand of the prograding dune. As a result, changes in the flow velocity were observed in the same places of the channel. These changes were well correlated with changes in the organic matter content and in the heavy-metal concentrations (Fig. 1).

## Modes of River-to-Floodplain Heavy-Metal Transfer

Heavy metals are transferred from a channel to the floodplain surface only when water table exceeds the bankfull stage. Because approximately 90 % or more of metal load can be associated with sediment particles, the pathways of metals to the floodplain are essentially the same as those of suspended sediments (Wyżga and Ciszewski 2010). Dissolved heavy metals are considered to play a minor role in the transfer of metals to the floodplain (Hostache et al. 2014). However, according to some estimates, even if the volume of water infiltrated into the floodplain were a few percentage points, it would correspond to approximately 10 % of the mass of pollutants deposited on the floodplain per a single flood event (Stewart et al. 1998). Although the prediction of infiltrated contaminants has been neglected in most chemical mass-balance studies, conditions that favour adsorption onto sediments can affect their entrapment (Gonzalez-Sanchis et al. 2015).

When flood water overtops the river bank, the contrast between the flow velocity in the channel and that in the floodplain produces eddies, which transfer solid particles and momentum from the deeper and faster flow in the channel to the shallower and slower flow over the floodplain (Knight and Shiono 1996). This mechanism, described by the diffusion mixing model, results in levee deposits, thickness and grain size of which decrease as the water-flow velocity slows down with an increasing distance from the channel (Pizzuto 1987). The other mechanism of sediment transfer by convection may occur where there is a component of flow perpendicular to the channel. Convection may result in strong differences in the amount of particles accumulated across the floodplain because the flow of water can transfer coarse particles on the floodplain by tractive movement (Marriott 1992). Sediment diffusion in lowland alluvial rivers results in the highest accumulation rate of metal load immediately at the river bank, whereas their maximum concentrations occur in thinner strata outside the levee zone if metal pollutants are primarily associated with finer size fractions. The sediment transfer by convection occurs in crevasse splays associated with a less uniform distribution of metal load across the floodplain and the highest metal concentrations behind the zone of coarse-grained deposits (Wyżga and Ciszewski 2010).

Convective sediment transport across the floodplain is particularly effective on sinuous rivers. River sinuosity induces helicoidal currents, which are responsible for the flood deposition of sandy fractions on the convex banks. For this reason, the thickness of sandy deposits can be higher on sinuous sections than on straight reaches modified by channel-training works (Ten Brinke et al. 1998). The strength of the helicoidal currents increases as discharge rises, and large floods produce lateral accretion deposits during point-bar formation in the near bank zone by both diffusion and convection processes (Hooke and Le 1975). These processes are active on natural rivers because stream-bank reinforcements reduce channel shifting on channelized ones.

## Floodplain Storage of Heavy-Metal Pollutants

The large variability of heavy-metal distribution in floodplains outlines the heterogeneous nature of sediment deposition. The maximal pollution may be concentrated in nearly isolated hotspots, discontinuous zones or strata, which decline markedly over distances of metres (Heaven et al. 2000; Ciszewski et al. 2008; Matys Grygar et al. 2014, 2016a). The most polluted sediments can be found in point-bar deposits of shifting channels and in natural levees when heavy metals are associated primarily with ore grains of high density (Marron 1989) or when they contain finer sediment laminae (Matys Grygar et al. 2016a). On rivers, where heavy-metal pollution is primarily associated with the finest size fractions, floods may create a thin but spatially extensive blanket of polluted overbank fines. Considerable pollution may also be stored in abandoned meanders (oxbow lakes) formed by cut-offs just before or during periods of pollution climax (Matys Grygar et al. 2016a).

Generally, the role of floods in the redistribution of polluted sediments within a river system is relative to its magnitude, and smaller river systems preserve the primary effects of large historical pollution for longer time. A closer look at the general trend of the fast longitudinal decrease in pollution usually shows a more complicated picture (Bird et al. 2008) and cannot be interpreted without a detailed and geomorphic description of individual rivers. The maximal heavy-metal concentrations may be lower in a floodplain than in a channel, but their decrease with the growing distance from the pollution source is usually less steep (Heaven et al. 2000; Macklin et al. 2003).

In formerly mined regions, a considerable portion of river systems was modified by extra sediment load inserted in the climax of mining. The associated heavy-metal concentrations could now be flattened and smeared by flood-induced reworking or preserved in terraces. Floodplain aggradation, usually enhanced during the mining period, was followed by erosion after mining cessation. In such cases, maximal pollution could be found in sediment bodies elevated above the current active floodplain (Brewer and Taylor 1997; Macklin et al. 1994; Miller et al. 1998). With time, persistent sediment reworking by floods decreases the extent of pollution with a growing distance from the former mines, and sometimes the current pollution maxima are further downstream from the original sources (Miller et al. 1998; Dennis et al. 2009; Foulds et al. 2014).

Particularly serious and spatially extensive floodplain pollution is caused by tailing dam failures or slurry remobilization, usually triggered by precipitation extremes (Hudson-Edwards 2003; Hudson-Edwards et al. 2001; Bird et al. 2008; Žák et al. 2009; Matys Grygar et al. 2014). Because such pollution events are associated with floods to which huge volumes of severely polluted slurry are inserted, these “pollution pulses” may produce widespread pollution peaks in sediment records (Resongles et al. 2014; Matys Grygar et al. 2014, 2016a), unless the river system has been severely polluted before the event (Hudson-Edwards 2003; Hudson-Edwards et al. 2001; Bird et al. 2008). The event layer can also be identified by different pollutant ratios (Resongles et al. 2014) or a specific isotope signature (Matys Grygar et al. 2014, 2016a).

If the floodplain part outside of a levee zone is regularly inundated by overbank flows, and if the river transports a sufficient amount of clay and silt fractions to produce yearly increments strata of at least several millimetres, sediment profiles with a stratigraphic order can be sampled there and then used as pollution archives (Grosbois et al. 2012; Nováková et al. 2013; Van Metre and Horowitz 2013; Dhivert et al. 2015b). The effect of sediment sorting on element concentrations must be taken into account to distinguish temporal and grain-size controls (Dung et al. 2013; Chen et al. 2014; Bábek et al. 2015). Geochemical normalization is more efficient for this purpose than conventionally used sieving (Kersten and Smedes 2002). Normalization considerably improves extraction of historical pollution signal from lithological variability inherent to fluvial systems and impacting also overbank fines. Sediment dating can be performed by means of the gamma spectrometry of fallout radionuclides or by a correlation of the rapid growth of metal concentrations or metal peaks in a series of several profiles with production characteristics known from the industrial history of a drainage basin (Ciszewski and Malik 2004; Lokas et al. 2010). Coarser (sandy) intercalations of extreme flood layers may also be used for their dating (Dhivert et al. 2015b; Zhang et al. 2015). In floodplains, it is necessary to distinguish overbank sequences from lateral channel deposits, the latter being less suitable for dating because of the contrasting rate and style of their deposition (Matys Grygar et al. 2013). The advantages of the sequences of overbank fines from the distal part of the floodplain as sedimentary archives are the relatively uniform stratigraphy, a lithology that prevents the vertical migration of pollutants in top strata, and a lower probability of erosion gaps (Lewin and Macklin 2003). The disadvantages of overbank sediment archives are the dilution of pollutants at overbank discharges (Nováková et al. 2013; Matys Grygar et al. 2013) and the risk of post-depositional processes due to reductimorphic processes or the physical translocation at depths closer to the groundwater table (Ciszewski et al. 2008; Du Laing et al. 2009). Furthermore, oxbow lake sediments may be valuable sedimentary archives. In them, the time of meander cut-off, either artificial (Van Metre and Horowitz 2013; Sedláček et al. 2016) or natural (Matys Grygar et al. 2016a), can be recorded as a lithological change; it provides a valuable time constraint for the younger sediments. Their deposition rate can be of the order of cm/y (Sedláček et al. 2016).

## Effect of Channel Engineering on Heavy-Metal Storage

Channel straightening by artificially cutting off river meanders is the most widespread modification of the river channel used to improve navigation and to obtain land for agriculture. Channel cut-offs are artificial features of the floodplain landscape resulting from intentional channel straightening. However, channel straightening may also occur naturally and produce oxbow lakes, particularly in river valleys with a low gradient. Artificial paleomeanders usually occur close to the channelized river and act during floods as efficient traps for sediment-associated heavy metals and other contaminants. The proximity of the river channel favours the rapid flood-related filling of cut-off channel segments, e.g. with 2-m deposits within less than 150 years, providing detailed records of the river pollution (Gocht et al. 2001). The filling processes are rapid, accurately reflecting changes in the river-sediment chemistry, as long as the channel cut-off is connected with the river (Bábek et al. 2008). When sediment is trapped only during flood episodes, the pollution record and metal concentrations are affected by the magnitude and frequency of overbank flows and the flow regulation and bed degradation following channelization (Dhivert et al. 2015a). Additionally, ponds may sometimes form on embanked river floodplains located in sites of historic dike breaches. These scars on the lower Rhine floodplain trapped as much as 6 m of fine, metal-polluted sediments over recent two centuries (Middelkoop 2000).

Channel training works associated with bank lining or groynes induces lateral channel stabilization, and channel narrowing, even by several times, expands the floodplain (Ciszewski and Czajka 2015). These processes usually disturb the natural regime of sediment transport because channel straightening causes an increase in the channel gradient and the rapid erosion of the riverbed and banks in the period immediately following the channelization works (Łajczak 2003). In channels cut in fine-grained alluvia, the rapid erosion of river banks can even led to increases in the channel width and the formation of a braided channel pattern (Kiss and Sipos 2007). A high rate of sediment accumulation on such an artificially shrunk floodplain is usually associated with channel degradation during channelization and can be even several times higher than before the channel-training period (Kiss et al. 2011). After the period of channel adjustment to new hydraulic conditions, the rate of overbank sand transfer onto the floodplain decreased, whereas finer grained sandy deposits with abundant clay layers became typical floodplain-accretion deposits, as observed on the Rhine River (Hesselink et al. 2003). Lateral channel fixation by bank reinforcements, which usually follow river straightening, confines the zone of lateral channel sediment accretion to the very narrow strip of land along channel banks, leading to the progressive channel narrowing and deepening. In lined and laterally stabilized channels, overbank deposition becomes the dominant process in the development of the floodplain (Ciszewski and Czajka 2015). Moreover, as the main floodplain-forming process, overbank deposition is also constricted by flood dykes to the narrow strip of the original floodplain, leading to a continuous increase in the floodplain elevation and the inherent, on many floodplains, decrease in the flooding frequency and sediment-accumulation rate (Hobo et al. 2014).

**Fig. 2:**
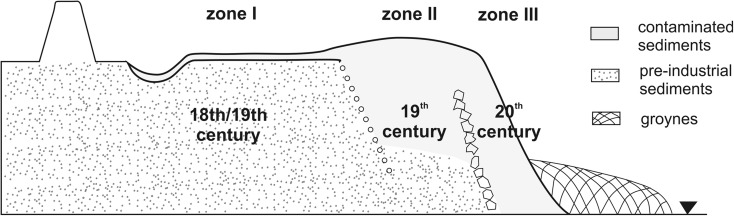
Distribution of metal-contaminated sediments in zones along the channelized reach of the Odra River is related to the width of the pre-regulations channel and repeated channel training works in 19^th^ and 20^th^ century (from Ciszewski and Turner 2009)

Groynes have been routinely constructed at the riverbanks of channelized European lowland alluvial rivers since the nineteenth century to direct the flow current to the channel centre and thus to enhance bed scour and to improve navigation conditions. Usually, groynes are directed at small angles against the flow direction to enable sediment entrapment (Sukhodolov et al. 2002). Groyne basins are known as sinks for fine, polluted sediments during low and average water stages, whereas during floods, they are sources of pollution (Baborowski et al. 2012), which is favoured by low compaction and large sediment thickness (Schwartz 2006). However, for example, in the historically contaminated Odra River, the groyne basins became long-term sinks for contaminated sediments because they operated as particularly effective sediment traps. With time, as the surfaces of the infill grew during high discharges, they were progressively keyed into the floodplain, resulting in a two- to threefold reduction in the channel width (Ciszewski and Turner 2009). Currently, groyne deposits in the incised, upper river reaches form approximately 4-m-thick sequences of fine sediments contaminated with heavy metals. The sediments contain black layers of coal particles intercalated with clean sands. The zone of these laminated deposits, which accumulate with a high average rate of approximately 5 cm/year, is confined to the width of the pre-regulation channel and does not exceed approximately 30 m on average (Ciszewski and Czajka 2015). In the middle river, reach stabilized by regulation structures, sediments contaminated with heavy metals occur in three zones of variable widths (Fig. 2). The zone of the nineteenth century groyne basins filled with nineteenth and twentieth century fine-grained sediments ranges in width from 10 to approximately 100 m, and the thickness of these deposits reaches 3 m. The most intensive polluted sediment accretion, with a rate of 5 cm/year in a 40-year period, has taken place in a river reach heavily polluted by mining and urban effluents (Ciszewski and Czajka 2015). This rate is among the maximum values observed for human-modified rivers (Provansal et al. 2010).

Generally, river channelization with groyne construction changes natural sediment cycling in a river valley, and in a polluted river, it produces a heavy-metal dispersal pattern different from that in non-engineered channels. This difference is reach specific and depends on the width of the pre-regulation channel, the length of groynes, the dynamics of the channelized river, the suspended load transported, the degree of river pollution and the length of the heavy pollution period. Heavy metal-contaminated deposits of the peak of the industrial era can be stratified. They contain a high organic-matter content, and commonly, parts of refuse material, e.g., bricks, plastic and ashes, can be called industrial alluvium (Ciszewski and Czajka 2015; Lewin 2013) or agro-industrial alluvium (Macklin et al. 2014). These deposits can also be characterized by a lack of bioturbation and upward fining or coarsening, and they usually accrete with the highest rate in groyne basins or in near-bank shelters other than those on the associated floodplain (Swennen and Van der Sluys 2002). They represent a novel, artificial sedimentary facies.

River embankments are perhaps the most widespread modifications in the river valleys of densely populated areas. Embankments confine flood-inundation zones to the relatively narrow strip of the original floodplain and reduce the area of overbank sediment accretion. These modifications are the most influential in river reaches conveying large amounts of sediment. In the upper Vistula River reach with the high sediment-accretion rate, the inter-embankment floodplain level is as much as 2 m higher above the surface outside embankments (Łajczak 1995). Rapid sediment deposition on the upper Vistula in the twentieth century was coeval with the peak of pollution, leading to the accumulation of thick sequences of sediments strongly polluted with heavy metals. Pollution with metals is two orders of magnitude lower in sediments of pre-industrial era behind flood dykes where zinc, cadmium and lead levels are close to the local geochemical background (Macklin and Klimek 1992). In most floodplains of lowland rivers draining industrialized areas, the average rate of sediment accretion decreases from approximately 1 cm in the proximity of the river channel to a few millimetres per year at flood dykes (Asselman and Middelkoop 1995; Kiss et al. 2011). For this reason, the thickness of the polluted sediments of the industrial era in the inter-embankment zone usually does not exceed several decimetres (Middelkoop 2002; Overesch et al. 2007; Ciszewski 2003). Generally, dykes seem to diminish the retention of pollutants by the shortening inundation time rather than to favour their conveyance losses because most dykes cut off depressions of the backswamp zone, where prolonged water stagnation with fine sediment deposition predominates.

## Storage of Heavy Metals in Dam Reservoirs

Dam reservoirs are traps for solid particulates (Van Metre and Horowitz 2013) and associated heavy metals (Palanques et al. 2014) transported through the river system. Globally, as much as 20–30 % of sediment transported by rivers is trapped in reservoirs, but this amount varies locally, primarily in relation to the reservoir depth, catchment topography and land use of the catchment (Vörösmarty et al. 2003; Syvitski et al. 2005). Reservoirs trap extremely variable parts of the annual metals load, which can reach 90 % for Pb, Cd and Cu (Schintu et al. 1991). Most deposits are accumulated in the shallow backwater zone, but the most polluted deposits usually occur in the deepest sections of reservoirs, where the undisturbed settling of the fine-grained clay and organic sediments takes place (Zhao et al. 2013). The deep parts of reservoirs where thermal stratification develops are characterized by oxygen depletion eventually leading to anoxic conditions, which triggers the reduction of nitrate, Fe and Mn hydroxides and sulphate (Friedl and Wüest 2002). Anoxic conditions in bottom sediments also cause the reduction and methylation of Hg, which enhances its export both as dissolved MeHg and bound in small organisms to remarkable distances downstream (Schetagne et al. 2009; Carrasco et al. 2011). Enhanced MeHg production immediately after the impoundment concerns all reservoirs, natural and artificial; however, the problem is particularly appealing when the sediments have already been polluted by Hg (Carrasco et al. 2011). Audry et al. (2010) described the conversion of labile Zn phases, silicates and oxides to sulphides, sulphates and Fe-oxide-associated species in the reservoir bottom. The reservoir sediment sinks are, however, not permanent: Changes in redox conditions (oxidation), acidification or physical disturbances, including flood inflows, can liberate the pollutants back to the water column (Coynel et al. 2007a, b; Audry et al. 2010; Yang et al. 2014; Hamzeh et al. 2014). At a large discharge or water release from the reservoirs, the unconsolidated sediments are easily re-suspended and turned back to the river system (Bi et al. 2014).

Floods and high river discharges may result in seasonal changes in heavy-metal concentrations in reservoir-bottom sediments, which document the transient nature of reservoir storage. Large floods, which flush sediment-associated heavy metals from polluted catchments, may increase the content of some metals in waters and sediments in the summer or during spring-melting periods (Kwapuliński et al. 1991; Szarek-Gwiazda et al. 2011). In the following part of a year, metals may be remobilized from the bottom and exit the dam lake (Kocharyan et al. 2003). In some years, fluxes of metals, mobilized from reservoir sediments, can be higher than metal loads entering that reservoir (Rzętała 2008). The enhanced heavy-metal pollution of bottom sediments is also observed in shallow reservoirs of heavily industrialized regions during dust emission in the cold season of the year (Reczyńska-Dutka 1985).

Dams tend to decrease the variation of fluvial discharges and to supress flooding and overbank deposition. Damming and discharge regulations prevent deposition in elevated surfaces in floodplains between extreme floods (Dhivert et al. 2015b) and makes deposition in low-lying surfaces more regular. Due to the decreased fluvial transport of solids, the pollution downstream from the dams may be less diluted by upstream, usually cleaner sediments (Van Metre and Horowitz 2013).

Dams change a spatial pattern of fluvial sediments exposed to the variation of redox conditions. The change in the ratios of risk elements, i.e., Pb and Zn on one hand and Fe on the other, in littoral areas subjected to regular (seasonal) water-level changes in a dam have recently been described (Liu et al. 2014). It is probable that prolonged waterlogging near the reservoir shore with a variable level will promote redox-driven processes and enhance mobility in a periodically or permanently inundated littoral zone.

The depth profiles in dam-reservoir sediments may be particularly valuable pollution archives (Audry et al. 2004; Sedláček et al. 2013). The sedimentary environment is much less variable in reservoirs than in floodplains and has much less reworking in deep, quiet locations. Their permanent existence under the water column, which decreases post-depositional migration, may provide more detailed reconstruction of historical pollution (Matys Grygar et al. 2012). Seismic profiles (Palanques et al. 2014) and other geophysical techniques (Bábek et al. 2008) are efficient in distinguishing reservoir sediments from pre-dam deposits. The onset of the reservoir deposition is a robust date point; otherwise, dam sediments can conveniently be dated by the gamma spectrometry of fallout radionuclides ^210^Pb and ^137^Cs (Sedláček et al. 2013, 2016). Other date points can be obtained by the assignment of coarser (sandier) intercalated sediments to extreme floods (Bábek et al. 2011; Dhivert et al. 2015b).

## Flood-Related Remobilization of Heavy Metals

Historically contaminated floodplains constitute a secondary source of river pollution. Its significance depends on the extent of physical and chemical processes operating on variable spatial and temporal scales (Macklin 1996). Proportions of the actual mechanisms at play are usually not distinguishable. The existence of many pollutant pathways varies depending on individual pollutants, catchment characteristics and changes with precipitation intensity and flood-wave discharge (Zonta et al. 2005; Coynel et al. 2007a, b; Resongles et al. 2015; Nováková et al. 2015).

Mobilization of sediment-associated heavy metals from floodplains is controlled by erosion during flood episodes, which on most of unregulated perennial rivers occur every 2 years, on average (Petit and Pauquet 1997 and references therein). Riverbank erosion is most intensive on natural, large meandering rivers. On low-gradient alluvial plains, the lateral channel shifts reach tens of metres per year, whereas on smaller rivers of lower energy, they may be of the order of decimetres to a few metres per year (Wang et al. 2014; Nicoll and Hickin 2010; Black et al. 2010). The erosive supply of the polluted sediment from riverbanks is reduced almost to zero by channel lining or revetment and is considerably suppressed by a variety of “softer” engineering measures, such as tree planting and channel dredging. In these and other laterally stable river reaches, floodplain erosion is limited almost solely to cultivated surfaces and paleochannels or depressions where high-water turbulence produces scars or chutes up to hundreds of metres long (Navrátil et al. 2008).

In areas of former metal mining, large amounts of pollutants may enter rivers through the flood-induced undercutting of waste tips exposed directly on banks (Foulds et al. 2014), the erosion of thick fine-grained floodplain sediment sequences (Dennis et al. 2003; Žák et al. 2009) or the erosion of paleochannels filled during the mining era, produced by the cut-off and abandonment of meander loops (Miller et al. 1998). In valleys with sparse vegetation in dry climates, a single, large flood may enlarge the river channel by two to three times and result in channel shifting by over 100 m. In such rivers, the erosion and redeposition of sediment-associated metal load is particularly effective along reaches characterized by low gradients and wide valley floors (Miller et al. 1999). Confined river reaches are net erosional during floods due to bank erosion (Thompson and Croke 2013) and act as transitional zones for sediment–associated heavy metals and other contaminants transported downstream (Macklin and Lewin 1989; Graf 1990). Metals associated with fine-grained sediments, stored in the alluvia of low-gradient reaches, are preferentially washed away as suspended sediment, even during small flood events, leaving behind coarser sediment as bed material (Žák et al. 2009). In small, severely polluted river reaches, metal loads in the suspended matter transported during floods may be very high, but their concentration always results from the relative contribution of historically polluted sediments and cleaner material eroded from cut banks, tributaries and catchment surfaces. Typically, the highest floods supply the largest loads of unpolluted sediments to the channel, whereas metal concentrations in suspended sediment tend to decrease with the increase in discharge (Salomons and Eysink 1981; Hutchinson and Rothwell 2008; Žák et al. 2009; Schultz-Zunkel and Krueger 2009). The dilution by cleaner sediments may, however, be insufficient to bring metal concentrations below target limits downstream from severely polluted areas (hotspots) where floods used to transport more polluted sediments (Matys Grygar et al. 2014; Dhivert et al. 2015a). The actual concentrations of pollutants within the flood wave are consequently a complex function of discharge and sediment supply (Resongles et al. 2015; Dhivert et al. 2015a). Due to different pollutant paths in historically polluted fluvial systems, the actual ratios of metal pollutants vary with the discharge (Resongles et al. 2015; Nováková et al. 2015).

**Fig. 3:**
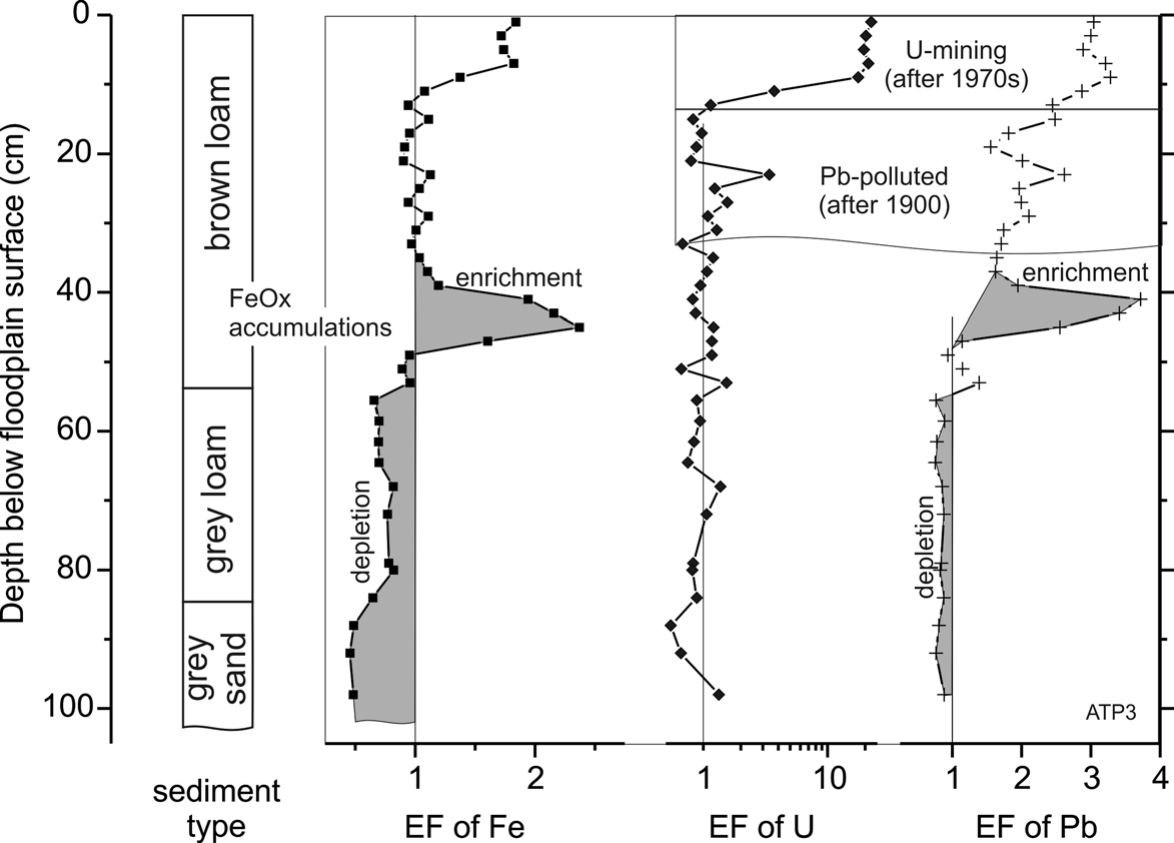
Depth profiles of Fe, U and Pb in redox-altered and polluted overbank sediments of the Ploučnice River, the Czech Republic; EFs (enrichment factors) are concentrations normalized to global (Fe) or local (Pb and U) background values (EF=1); Fe concentrations was normalized by Al concentrations and divided by mean upper crustal global reference Fe/Al ratio; U and Pb concentrations were divided by local background concentrations for the overbank sediments of the studied river (according to Matys Grygar et al. 2014; Matys Grygar et al. 2016a)

The chemical remobilization of metals during floods is related to progressive oxygen depletion by microbial and root respiration during floodplain inundation. With the change from aerobic to reductive conditions, the reductive dissolution of Fe and Mn hydroxides takes place, and it is also controlled by pH, salinity, organic matter content and temperature (Rinklebe and Du Laing 2011). In reducing conditions, metals such as Fe and Mn and commonly associated pollutants such as As, Cd, Cr, Mo, Ni and Zn can be released from the solid phase to pore waters (Shaheen et al. 2014; Hindersmann and Mansfeldt 2014). However, mobile fractions of metals are not simply transported to water bodies, and metal transfer is not currently quantifiable (Schultz-Zunkel and Krueger 2009). Recently, diffusive gradient in thin films (DGT) technique is used to determine pore water profiles and remobilization of heavy metals at the sediment/water interface (Wu et al. 2016). Flood recession followed by the drying and aeration of floodplain soils reverses the processes of metal dissolution. In an oxic environment, Fe and Mn re-precipitate as oxides and scavenge heavy metals back to the solid state (Du Laing et al. 2009). This phenomenon is shown in depth profiles of overbank sediments of the Ploucnice River in the Czech Republic (Fig. 3). Iron and Pb are depleted in reduced (grey coloured) strata and accumulated in Fe-oxide rich concretions near boundary of reduced and unaltered (brown coloured) overbank fines (Matys Grygar et al. 2014, 2016a). Short-term flood episodes do not affect pH and have lesser effect on metal mobilization than redox changes. Moreover, it could be expected that single and rapidly flowing flood waves have a minor effect on the metal migration within floodplain sediment profiles, but it was shown that in areas with a longer flooding duration, the mobility of some metals, expressed by their speciation, was higher than in areas inundated less frequently (Shaheen and Rinklebe 2014). Vertical metal displacement is favoured by the frequent fluctuation of water-table levels and, in addition to the visible accumulation of the secondary Fe and Mn oxyhydroxides, may manifest in mineral breakdown and pseudomorphing or high levels of exchangeable and specifically adsorbed metals (Hudson-Edwards et al. 1998). The consequences of past redox changes are easily revealed as “erratic” Fe and Mn depth profiles in floodplain-sediment cores and systematic depletion at depths below the common water table (Matys Grygar et al. 2013). The translocation rate of metals can be high in an acidic floodplain environment, and mobile metals can retain in less acidic zones abundant in Fe oxides (Kraus and Wiegand 2006), clay-rich zones (Cappuyns and Swennen 2004) or organic-rich zones, giving peaks in vertical metal distribution (Ciszewski et al. 2008). Metal redistribution with migrating groundwater has been observed particularly in profiles of coarse-grained deposits, which exhibit anomalous metal peaks at levels related to the depth of the most frequent water-table fluctuation (Taylor 1996; Ciszewski et al. 2008; Matys Grygar et al. 2013). Although fluxes of heavy metals with groundwater to a river can be extremely variable locally and change over an order of magnitude at distances of hundreds metres (Coynel et al. 2007a, b), their role in natural systems is likely minor compared with physical remobilization by fluvial erosion. Floodplains as a diffusive source of heavy metals, are undoubtedly most important during floods and high-water stages (Aleksander-Kwaterczak and Ciszewski 2012; Palumbo-Roe et al. 2012).

## Mapping Floodplain Pollution by Heavy Metals

**Fig. 4:**
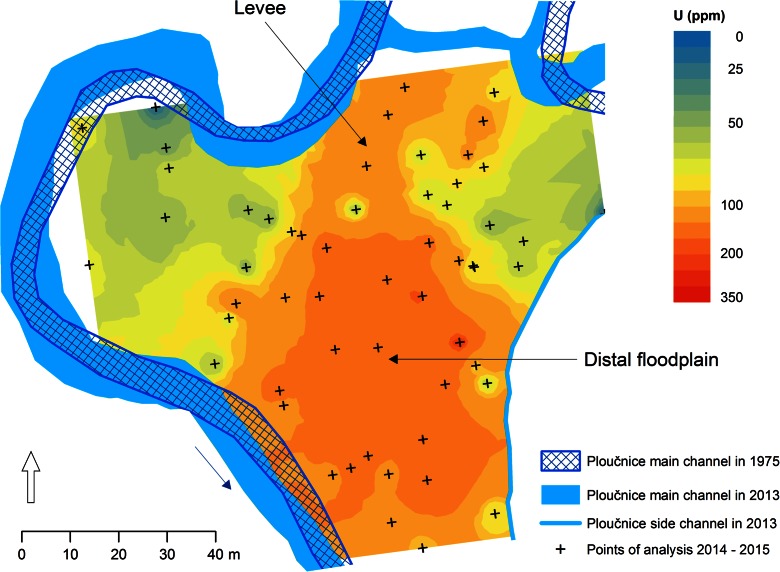
Distribution of U in top 0–10 cm in floodplain of the Ploučnice River, typical for rivers dominated by metal pollution of fine grained sediments mixed with unpolluted parent coarser grained sediment. Data obtained by in situ (portable, handheld) XRF mapping. Lower concentrations of U (<90 ppm) are in coarser sediments in areas covered by less polluted post-mining sediments in proximal floodplain, higher concentrations are in finer distal floodplain deposits and in certain parts of levee (J. Elznicová, unpublished results)

The spatial distribution of heavy-metal pollution in floodplains has been documented by many authors (Macklin et al. 1994; Miller et al. 1998; Heavens et al. 2000; Notebaert et al. 2011; Hobo et al. 2014; Foulds et al. 2014). Investigations indicate that an extensive sampling using depth profiles (sediment cores) is required to faithfully describe it, and it is best if the coring sites are selected on the basis of geomorphological descriptions. Detailed three-dimensional pollution mapping is needed along embanked rivers (Hobo et al. 2014), along rivers with significant natural or artificial channel shifts (Ciszewski and Malik 2004; Matys Grygar et al. 2013; Ciszewski et al. 2014; Foulds et al. 2014), and particularly on floodplains with a temporally variable aggradation/erosion balance (Macklin et al. 1994; Miller et al. 1998). In floodplains with a more complex microtopography and pollution history, heavy metals may be concentrated in hotspots, the position of which is hardly predictable (Heaven et al. 2000; Miller and Orbock- Miller 2007; Matys Grygar et al. 2014; Fig. 4). Extensive sampling is also required in catchments with variable geology and geomorphology (Amorosi and Sammartino 2007; Peh et al. 2008; Amorosi et al. 2014; Matys Grygar et al. 2016B) and numerous pollution sources along the river course (Matys Grygar et al. 2013, 2016b).

A faithful description of historically polluted floodplains may require sampling the entire thickness of floodplain fines (Lecce and Pavlowsky 2014; Matys Grygar et al. 2013, 2014, 2016a). The average vertical deposition rate of overbank fines by a medium-sized river is of the order of millimetre per year, and in-channel sedimentation, including oxbow lakes, may be up to a few centimetre per year (Sedláček et al. 2016). Century-old polluted strata can hence be expected at depths between a few centimetre to a few metre, the latter being particularly probable in the channel belt (Matys Grygar et al. 2013, 2016b). If erosion basis and/or floodplain level changed in or since the pollution period, each geoform in floodplain should be examined (Macklin et al. 1994; Miller et al. 1998). To describe the internal floodplain architecture, Houben (2007) recommended a series of drill cores across the floodplain with spacing less than the current channel width. In polluted floodplains, that approach should be applied at least to the range of channel shifts during the pollution period and the nearby floodplain (Notebaert et al., 2011; Matys Grygar et al. 2013). Because the thickness of polluted strata can also vary for a single flood deposit from the order of mm to the massive, decimetres thick laterally deposited body, sampling in cores should be continuous with steps in the order of cm.

Any analytical method can be used for pollution mapping if it is sufficiently productive. AAS, ICP MS or ICP OES and X-ray fluorescence spectroscopy (XRF) are currently the most popular methods, employed by many commercial laboratories. To enhance the analytical productivity at low costs, there is a tendency to replace methods that require time- and cost-demanding dissolution steps (total decomposition with mixed acid digestion or melting) by XRF with pressing or only pouring samples to measuring cells (Matys Grygar et al. 2014; Perroy et al. 2014). To get around the total decomposition, pseudototal decay (*aqua regia* or single acids) has been proposed and is still used. However, it is not suitable to quantify most lithogenic elements suitable for geochemical normalization.

Several types of portable instruments are suitable for direct pollution mapping (Gałuszka et al. 2015; Horta et al. 2015), but they are primarily tested for soils and industrial, mining and waste disposal sites. Portable XRF (PXRF) spectrometers are particularly promising for pollution studies: In situ analyses require no sampling/sample pre-treatment and can easily produce pollution maps (Hürkamp et al. 2009), identify pollution hotspots (Weindorf et al. 2012) and instantaneously produce information essential for decision making in technological operations (Lemiere et al. 2014). The determination limits of PXRF are usually sufficient for elements such as Pb and Zn, for elements such as As, Cu and Ni in moderately polluted sediments, and for most risk elements in severely polluted sites. Varying humidity, organic matter content and texture effects may be a hindrance, the handling of which requires empirical corrections and further work (Lemiere et al. 2014; Weindorf et al. 2014).

Other methods for direct in situ mapping are less common. For radioactive pollution, gamma-activity mapping should be the method of the first choice (Martin et al. 2015). In situ gamma spectrometry can also produce lithological mapping in floodplains (Spadoni and Voltaggio 2013). Attempts have been made to use Vis-NIR spectrometers (VNIR) with empirical calibration for soil- or floodplain-pollution mapping. The expectations have been based on the successful correlation of heavy-metal concentrations with the concentration of their main carriers, Fe-oxides, organic matter and clay minerals obtained by laboratory VNIR measurements (Kooistra et al. 2001, 2003). The hindrance for in situ mapping is still the problem of varying humidity and the variable state of the vegetation in the land cover.

## Summary

Floods play a crucial role in the remobilization of heavy metals from historically polluted deposits, whereas present-day pollutants are primarily transported during moderate and low flows. Moreover, floods, which normally represent only a small percentage of annual discharge, are a phenomenon that creates and reshapes the floodplain and is responsible for the transfer of metal pollutants from temporary sinks in the channel. In perennial rivers, metals in dissolved forms or associated with fine sediments can be transported over long distances depending on the flow competency, whereas sediments can be more persistently stored in a floodplain at overbank flows. For this reason, the length of the river and the degree of its modification, including structures such as groynes, weirs, dams, oxbow lakes or side channels, which are important traps for fine sediments, control the pollutant conveyance through the river system. The river damming and variety of engineering measures in floodplains suppress the effect of floods and limit overbank processes in most rivers.

Floods control the distribution of sediment-associated heavy metals in alluvial channels by the creation of mezoforms, which are then modified during moderate and low flows and filled or infiltrated with polluted fines. Sediment sorting in a channel during both floods and lower discharges and in a floodplain during floods is a highly important mechanism responsible for rapid changes in heavy-metal concentrations in sedimentary sequences. The variable sorting overlaps the record of the historical changes of river pollution, and all reconstructions of heavy-metal pollution from those sequences must correct concentrations for grain-size effects.

Proportions of flood-related physical and chemical metal mobilization from historically contaminated floodplains to rivers are hardly quantifiable because they are so site specific. However, both processes are most intensive during certain stages of floods. Typically, the physical remobilization of metals seems to dominate over leaching under high discharges in reaches where fine-grained alluvia are easily eroded. By contrast, floodplains constitute diffusive metal sources even in laterally stable reaches, particularly if polluted fines occur in a framework of coarse-grained sediments favouring the migration of dissolved metals. The relative contribution of physical and chemical metal mobilization depends not only on the rate of river erosion and the average grain size of alluvia but also on the depth and frequency of water-table fluctuations.

Flood-related metal storage and remobilization are controlled by river channelization, but their influence depends on the timing and extent of the engineering works. Generally, the accretion of polluted sediments in groyne basins and in cut-off channels, performed not long before the pollution period, makes them hotspots of pollutants transported in enhanced amounts during high-water stages. Moreover, floodplains of lined river channels that adjust to new, post-channelization hydraulic conditions become the permanent sink for fine and polluted sediments, which accumulate solely during overbank flows. In such laterally stable river reaches, metal mobilization occurs only by slow leaching, and their sediments, which accrete at a moderate rate, are the best archives of the catchment pollution with heavy metals.
